# Improving the Assessment and Management of Sleep Problems in a Specialist NHS Gambling Treatment Service

**DOI:** 10.1192/bjo.2024.565

**Published:** 2024-08-01

**Authors:** Harry Fagan, Holly Austin, Mat King, Samuel Chamberlain, Konstantinos Ioannidis

**Affiliations:** 1 Southern Gambling Service, Southern Health NHS Foundation Trust, Southampton, United Kingdom; 2 Clinical and Experimental Sciences (CNS and Psychiatry), Faculty of Medicine, University of Southampton, Southampton, United Kingdom; 3 Dorset HealthCare University NHS Foundation Trust, Poole, United Kingdom

## Abstract

**Aims:**

Sleep disorders, such as insomnia, are common in the general population and in patients with psychiatric conditions including the behavioural addiction Gambling Disorder (GD). The NHS Southern Gambling Service (SGS) is a tertiary centre providing evidence-based assessment and treatment for people affected by GD across the South-East of England. We aimed to assess the prevalence of sleep problems in help-seeking adults with gambling difficulties, including the association with gambling severity and other measures of psychopathology, and determine if 1) sleep is appropriately assessed and 2) whether sleep disorders are appropriately diagnosed and managed, in line with NICE guidelines, in this particular cohort.

**Methods:**

All patients referred from September 2022–October 2023 who completed an initial clinician assessment were included. Gathered data included age, gender, pre-existing physical health conditions, and scores from the following questionnaires: Gambling Symptoms Assessment Scale (GSAS), Pathological Gambling Yale-Brown Obsessive Compulsive Scale (PG-YBOCS), Brief Pittsburgh Sleep Quality Index (B-PSQI), Patient Health Questionnaire (PHQ-9) and Generalised Anxiety Disorder 7 (GAD-7). Data analysis was performed under ethical approval (23/HRA/0279). Relationships between gambling severity and sleep quality, and depressive/anxiety symptoms were explored (using Pearson correlation coefficient). In patients with a B-PSQI score > 5 (suggestive of underlying sleep disorder), we determined whether sleep problems were appropriately assessed and managed.

**Results:**

83 patients completed an initial clinician assessment (81% male, average age 38 years). Baseline B-PSQI scores were weekly positively correlated with gambling severity on the GSAS (r = 0.18) and the PG-YBOCS (r = 0.10) and anxiety symptoms severity on the GAD-7 (r = 0.26). Baseline B-PSQI scores were moderately positively correlated with depressive symptom severity on the PHQ-9 (r = 0.39) and higher B-PSQI scores were noted in patients reporting suicidality.

54/83 (65%) patients had a baseline B-PSQI score > 5, of these, seven (13%) had a clearly documented management plan for insomnia in line with NICE guidelines.

**Conclusion:**

Most patients referred to SGS had baseline B-PSQI scores suggestive of current sleep problems. B-PSQI scores were positively correlated with gambling severity and severity of anxiety and depression. Findings highlight that sleep problems are common in people presenting to the NHS gambling service, but also that there is scope to improve and extend signposting for affected individuals to receive sleep-specific support. The audit findings have been presented to the SGS team; resources for the assessment and management of sleep problems have been shared and a re-audit is planned for Summer 2024.

Additional authors: Dr. Jodi Pitt, Esther Gladstone, Dr. Peter Hellyer.

